# Fzr/Cdh1 Promotes the Differentiation of Neural Stem Cell Lineages in *Drosophila*

**DOI:** 10.3389/fcell.2020.00060

**Published:** 2020-02-11

**Authors:** Phuong Thao Ly, Hongyan Wang

**Affiliations:** ^1^Neuroscience & Behavioral Disorders Programme, Duke-NUS Medical School, Singapore, Singapore; ^2^Department of Physiology, Yong Loo Lin School of Medicine, National University of Singapore, Singapore, Singapore; ^3^NUS Graduate School for Integrative Sciences and Engineering, National University of Singapore, Singapore, Singapore

**Keywords:** NSC, neuroblast, GMC, differentiation, Fzr/Cdh1

## Abstract

How stem cells and progenitors balance between self-renewal and differentiation is a central issue of stem cell biology. Here, we describe a novel and essential function of *Drosophila* Fzr/Cdh1, an evolutionary conserved protein, during the differentiation of neural stem cell (NSC) lineages in the central nervous system. We show that Fzr, a known co-activator of Anaphase Promoting Complex/Cyclosome (APC/C) ubiquitin ligase, promotes the production of neurons from neural progenitors called ganglion mother cells (GMCs). However, knockdown of APC/C subunit *Ida* or another APC/C co-activator *CDC20* does not similarly impair GMC-neuron transition. We also observe a concomitant loss of differentiation factor Prospero expression and ectopic accumulation of mitotic kinase Polo in *fzr* mutant clones, strongly supporting the impairment of GMC to neuron differentiation. Besides functioning in GMCs, Fzr is also present in NSCs to facilitate the production of intermediate neural progenitors from NSCs. Taken together, Fzr plays a novel function in promoting differentiation programs during *Drosophila* NSC lineage development. Given that human Fzr is inactivated in multiple types of human cancers including brain tumors and that Fzr regulates neurotoxicity in various models of neurodegenerative diseases, our study on the role of Fzr in turning off proliferation in neuronal cells may provide insights into how Fzr deficits may contribute to human neurodegenerative diseases and tumors.

## Introduction

Understanding how stem cells maintain their self-renewal capacity and how their progeny differentiate into specific fates are essential to comprehend developmental processes as well as to exploit the therapeutic potential of stem cells for regenerative medicines and cancer treatments. The *Drosophila* larval brain NSCs, or neuroblasts, have emerged as a fertile model for studying stem cell self-renewal and differentiation *in vivo* (Li et al., 2014). In the *Drosophila* central nervous system, type I NSCs divide asymmetrically to self-renew and to generate a smaller daughter cell called the GMC that only divides once to give rise to two terminally differentiated neurons or glial cells (reviewed in Homem et al., 2015). Besides type I NSCs, 8 type II NSCs located bilaterally in the central brain divide asymmetrically to self-renew and generate intermediate neural progenitors (INPs), which can divide 4–6 times to give rise to GMCs and neurons (Bello et al., 2008; Boone and Doe, 2008; Bowman et al., 2008). Newly generated INPs need to differentiate into mature INPs before division. The transcription factor Earmuff (Erm), a homolog of the vertebrate Forebrain embryonic zinc-finger family, is required for INP maturation (Bowman et al., 2008; Weng et al., 2010). Erm works together with the SWI/SNF chromatin-remodeling complex Brahma (Brm) in immature INPs to restrain the developmental potential of INPs (Eroglu et al., 2014; Koe et al., 2014; Janssens et al., 2017; Liu et al., 2017). Several studies in both mammals and *Drosophila* have indicated that neuronal differentiation is actively maintained. To date, only a few factors have been implicated in maintaining *Drosophila* neuronal differentiation. These factors include the homeodomain transcription factor Prospero (Pros, human homolog Prox1) (Caussinus and Gonzalez, 2005; Choksi et al., 2006; Maurange et al., 2008), RNA splicing factor Midlife Crisis (Mlc) (Carney et al., 2013), BTB-zinc finger transcriptional factor longitudinals lacking (Lola) (Southall et al., 2014), and the zinc finger transcription factor Nervous fingers 1 (Nerfin-1) (Froldi et al., 2015). In the absence of Pros, GMCs revert to NSCs instead of committing to differentiation (Betschinger et al., 2006; Choksi et al., 2006; Lee et al., 2006). Mlc regulates expression and splicing of Pros and the loss of Mlc leads to the accumulation of ectopic NSC-like cells originating from dedifferentiated neurons, but these NSC-like cells are stalled during cell cycle and do not form tumors (Carney et al., 2013). Lola, on the other hand, acts redundantly with Pros. The loss of Lola is associated with neuron-to-NSC reversion and tumorigenesis in the optic lobes, but not in the central brain due to the compensatory functions of Pros (Southall et al., 2014). Recently, Nerfin-1 has been shown to function downstream of Pros to maintain neuronal differentiation. Neurons in *nerfin-1* mutants first increase their cellular size, then switch off neuronal program and start to express the NSC-identity program (Froldi et al., 2015). However, it remains elusive whether other cellular factors play a role in differentiation of NSC lineages.

Fzr (fizzy-related) or Rap (retina aberrant in pattern) or Cdh1, the *Drosophila* homolog of mammalian FZR1, is an evolutionarily conserved protein that functions as a positive regulator of Anaphase-Promoting Complex/Cyclosome (APC/C), which regulates cell-cycle progression via ubiquitin-mediated proteolysis (Pines, 2011). While APC/C interacts with CDC20/Fizzy (Fzy) to mediate chromatid separation during metaphase-to-anaphase progression and drive mitotic exit, APC/C binds to Fzr/Cdh1 during mitotic exit and G1 phase to conclude mitotic exit as well as to participate in non-mitotic functions such as endoreplication, quiescence, and differentiation (Eguren et al., 2011). In *Drosophila*, Fzr has also been shown to promote mitotic exit (Meghini et al., 2016) and to regulate various non-mitotic functions, including glial migration (Silies and Klämbt, 2010) and glial cell number (Kaplow et al., 2008), synapse size and activity at neuromuscular junctions (van Roessel et al., 2004), terminal mitosis (Reber et al., 2006), endocycle and endoreplication (Sigrist and Lehner, 1997; Schaeffer et al., 2004; Narbonne-Reveau et al., 2008; Djabrayan et al., 2014), and in retinal differentiation (Martins et al., 2017). However, how Fzr functions in NSC lineages remains unknown.

Here, we describe a novel and essential role for *Drosophila* Fzr in GMC-to-neuron transition in both type I and type II NSC lineages of the developing larval brains. In *fzr*^–^ mutant clones, GMC population expands at the expense of neurons. We also observe a concomitant loss of Pros expression and ectopic accumulation of Polo in mutant clones, suggesting the impairment of GMC to neuron differentiation. The localization of EGFP-Fzr^BAC^ in late  GMCs is consistent with its novel function in GMCs. Moreover, *Fzr* also regulates NSC to INP transition, through genetic interaction with *Brm* and *Erm*.

## Materials and Methods

### Fly Stocks and Genetics

FRT19A, *fzr*^A^ (#52384), FRT19A, *fzr*^B^ (#52385), and Dp(1;3)DC120 (#30265) were obtained from BDSC. FRT19A, *fzr^G0418^* (#111943) was obtained from Kyoto Stock Center. *UAS-Fzr-HA* (#F000893) was obtained from FlyORF. *UAS-HA-Rca1* is a gift from Dr. Frank Sprenger (Grosskortenhaus and Sprenger, 2002) and FRT19A, *fzr^8F3^* (P{neoFRT}19A/FM7) was a gift from Dr. Christian Klambt. EGFP-Fzr^BAC^ was generated in this study.

RNAi lines used in this study: *erm*_RNAi (BDSC #26778), *fzr*_RNAi (GD#25553), *cdc20*_RNAi_1 (GD#40500), *cdc20*_RNAi_2 (GD#44834), *ida*_RNAi (BDSC#34522); *brm*_RNAi (GD#37720), β*-gal*_RNAi (BDSC#50680), and *bcd*_RNAi (GD#48966).

Neural stem cell drivers included *insc*-Gal4 (BDSC#8751; 1407-Gal4) or *wor*-Gal4 (BDSC#56553). Glial driver was *repo*-Gal4 (BDSC#7415). Type I NSC driver (*ase*-Gal4; UAS-mCD8-GFP, T. Lee). Type II NSC driver (w; UAS-Dicer2, *wor*-Gal4, *ase*-Gal80; UAS-mCD8-GFP) (Bowman et al., 2008). INP driver (*erm*-Gal4/CyO) (Pfeiffer et al., 2008). Other drivers used in this study are: *nerfin-1*-Gal4, UAS-mCD8-GFP (Louis Y. Cheng), *pros*-Gal4 (BDSC#80572), and *elav*-Gal4 (BDSC#458). UAS-Dcr2 (BDSC#24650) or/and UAS-CD8-GFP (BDSC#32186) was used together with various Gal4 drivers or RNAi stocks.

Experiments with mutants were performed at 25°C, and experiments for RNAi-mediated knockdown or overexpression were carried out at 29°C.

### Clonal Analysis

MARCM clones were generated as previously described (Lee et al., 1999). Briefly, the larvae were heat-shocked twice at 37°C for 2 hours (h) each, shortly after larval hatching (ALH) and at 10–16 h after the first heat shock. Larvae were further aged for another 3 days at 25°C before dissection.

MARCM driver used is w *hsFLP, FRT19A, tubP-Gal80; UAS-nlsLac, UAS-mCD8-GFP; tub-Gal4* (Lee et al., 1999).

### Immunochemistry

Wandering third instar larval brains were dissected in cold PBS and fixed in 4% EM-grade formaldehyde in PBS at room temperature (RT) for 22 minutes (min), following by three washes in 0.3% Triton-X in PBS (PBST). The sample was incubated with blocking buffer (3% BSA in PBST) for 45 min at RT, followed by incubation with primary antibody mixture overnight at 4°C. After three washes in PBST, larval brains were incubated with secondary antibody mixture for 90 min at RT, followed by two washes, and mounted in a glycerol based mounting medium (10 mg/ml of *p*-Phenylenediamine in PBS, 1:10 dilution with glycerol). For staining of DNA, additional incubation of the sample with diluted Topro-3 in PBST was performed just before adding mounting medium. Samples were imaged with Zeiss LSM 710 confocal microscopy and images were processed with Zeiss black software.

The following antibodies were used in this study: guinea pig anti-Dpn (1:1000, J. B. Skeath), rabbit anti-Ase (1:1000, Y. N. Jan), rabbit anti-Repo (1:500, W. Chia), rat anti-CD8 (1:250, Life technologies, Cat#MCD0800), rabbit anti-PH3 (1:200, Sigma, Cat#H9908-25UL: AB_260096), rat anti-Elav (1:40, DSHB, Cat#Rat-Elav-7E8A10), guinea pig anti-Nerfin-1 (1:1000, Louise Y. Cheng), mouse anti-Miranda (1:40, F. Matsuzaki), rabbit anti-aPKC ζ C20 (1:100, Santa Cruz Biotechnology Cat# sc-17781), guinea pig anti-Baz (1:500; A. Wodarz), mouse anti-Pros [1:10, DSHB, Cat# Prospero (MR1A)], rabbit anti-Polo (1:100, C. Sunkel), rabbit anti-GFP (Molecular Probes, Cat#A21311, 1:500), mouse anti-α-tub (1:200, Sigma-Aldrich, Cat#T6199-200UL), rabbit anti-β-gal (1:100, Invitrogen, A-11132), mouse anti-β-gal (1:1000, Promega, Cat#Z3781), rat anti-HA (1:2000; Roche, Cat #11867423001, Clone 3F10), rabbit anti-PntP1 [1:500, J. B. Skeath (Alvarez, 2003)]. DNA was labeled by ToPro-3 (1:5000, Invitrogen, Cat#T3605).

The secondary antibodies (Alexa 647, Alexa 568, and Alexa 488 or Alexa 405) were obtained from Jackson Immuno Research Laboratories, Inc. and used at 1:500 dilution. Phalloidin 488 (1:500, Invitrogen^TM^, A12379), was added together with secondary antibodies.

### Generation of Transgenic Flies

BAC line (CH321-61L05) was used to generate *EGFP-Fzr^BAC^* genomic construct according to the method described previously (Zhang et al., 2019). Briefly, a fragment containing enhanced GFP (EGFP) tag and Kanamycin-resistant gene was amplified from PL-452 EGFP vector and inserted to the N-terminus of *fzr* coding sequence by recombination using SW102 electrocompetent cells. The resulting cassette was then electroporated into *ara*-inducible Cre carrying SW106 electrocompetent bacteria to remove the Kanamycin resistant gene. The correct clone carrying EGFP-Fzr^BAC^ was electroporated into EPI300 cells for amplification and the BAC DNA was purified using BACMAX kit (Epicentre Biotechnologies Cat# BMAX044). The *EGFP-Fzr^BAC^* was sent to BestGene, Inc. for injection into y^1^ w^67^c^23^; P{CaryP}attP40 (estimated cytosite 25C6) background. The primers used for recombination are listed in Supplementary Table 1.

### Statistical Analysis

GraphPad Prism 6 software was used for statistical analysis. All data were presented as mean ± SD. Unpaired two-tail *t*-tests were used for two sample comparisons and one-way ANOVA for comparison of more than two groups. In ANOVA, Dunnett’s *post hoc* test was used to obtain the *P*-values for pairwise comparison. In this work, comparisons were performed against wild-type or control, unless otherwise indicated by a line between two genotypes. A value of *P* ≤ 0.05 was considered as statistically significant, ^∗^ indicated *P* ≤ 0.05, ^∗∗^ indicates *P* ≤ 0.01, ^∗∗∗^ indicates *P* ≤ 0.001, and ^****^ indicates *P* ≤ 0.0001. *P* > 0.05 was considered as statistically non-significant (ns).

## Results

### Loss of Fzr in NSC Lineages Results in Ectopic Progenitor GMCs

**FIGURE 1:**
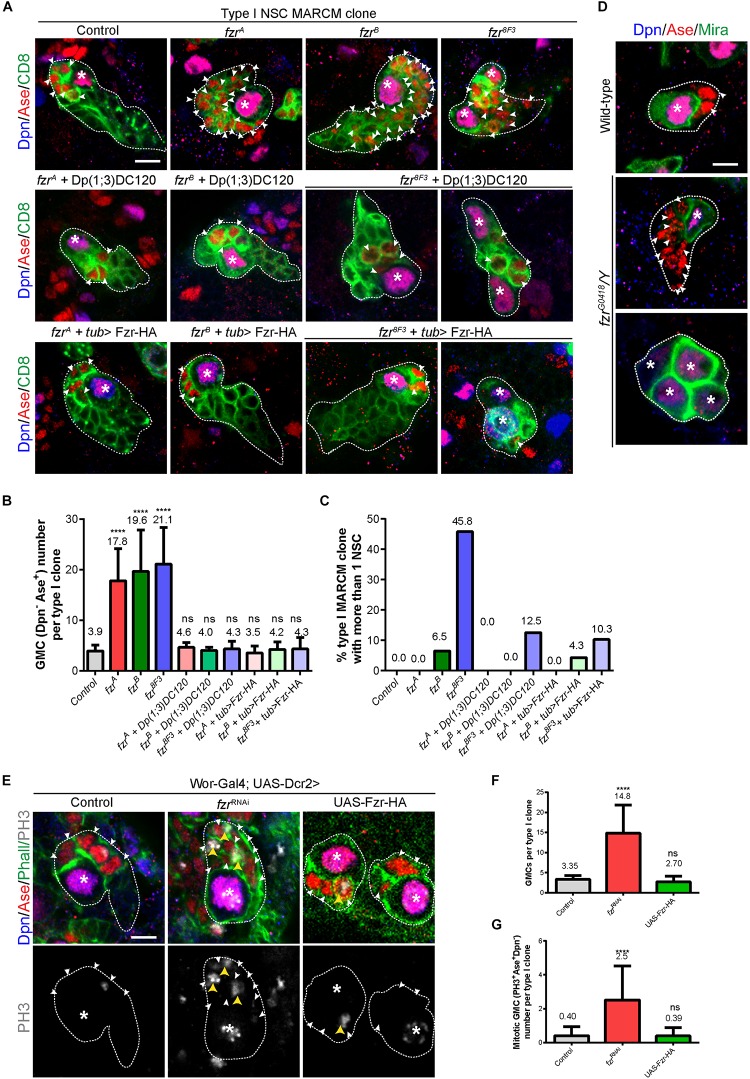
Ectopic GMCs are generated upon Fzr deletion. **(A)** Type I NSC clones of MARCM driver control (FRT19A), *fzr*^A^, *fzr*^B^, *fzr^8F3^*, *fzr*^A^ + Dp(1;3)DC120, *fzr*^B^ + Dp(1;3)DC120, *fzr^8F3^* + Dp(1;3)DC120, *fzr*^A^ + *tub* > *Fzr-HA*, *fzr*^B^ + *tub* > *Fzr-HA*, and *fzr^8F3^* + *tub* > *Fzr-HA* were labeled with Dpn, Ase and CD8. *tub* > *Fzr-HA* refers to *UAS-Fzr-HA* driven by *tub-Gal4* from MARCM driver, so Fzr-HA is expressed only in MARCM clones that lose *tub-Gal80* upon mitotic recombination. **(B,C)** Quantifications of GMC number **(B)** per type I MARCM clones and the percentage of MARCM clones with two or more NSCs **(C)** for **(A)**. (Control, *n* = 40; *fzr*^A^, *n* = 46; *fzr*^B^, n = 46; *fzr^8F3^*, *n* = 24; *fzr*^A^ + Dp(1;3)DC120, *n* = 23; *fzr*^B^ + Dp(1;3)DC120, *n* = 20; *fzr^8F3^* + Dp(1;3)DC120, *n* = 16; *fzr*^A^ + *tub* > *Fzr-HA*, *n* = 31; *fzr*^B^ + *tub* > *Fzr-HA*, *n* = 23; *fzr^8F3^* + *tub* > *Fzr-HA*, *n* = 29. **(D)** Type I NSC lineages of wild-type and hemizygous *fzr^G0418^*/Y larvae were labeled with Dpn, Ase, and Mira. Each genotype, *n* > 30 clones in at least five different brain lobes. **(E)** Type I NSC lineages of control (β*-gal^RNAi^)*, *fzr^RNAi^*, or UAS-Fzr-HA with NSC-driver (*wor*-Gal4; UAS-Dcr2) were labeled with Dpn, Ase, Phall (Phalloidin, cortical actin), and PH3. **(F,G)** Quantifications of the number of GMCs **(F)** and mitotic GMCs **(G)** per type I clones for **(E)**. For **(F)**, control, *n* = 20; *fzr^RNAi^, n* = 50; *UAS-Fzr-HA*, *n* = 56. For **(G)**, control, *n* = 50; *fzr^RNAi^, n* = 50; *UAS-Fzr-HA*, *n* = 41. Data are presented as mean ± SD. ****P ≤ 0.0001. Asterisks, NSCs; white arrowheads, GMCs; yellow arrowheads, mitotic GMCs; white dotted lines, clone outline. Scale bars: 5 μm. n, number of quantified clones. Ase, Asense; Dcr2, Dicer 2; Dpn, Deadpan; Fzr, Fizzy and cell division cycle 20 related; GMC, Ganglion Mother Cell; MARCM, mosaic analysis with a repressible cell marker; Mira, Miranda; ns, statistically non-significant; NSC, neural stem cell; Phall, Phalloidin; PH3, phospho-Histone H3; UAS, upstream activating sequence; Wor, Worniu.)

In order to evaluate the possible function of Fzr in NSC lineage development, we generated mosaic analysis with a repressible cell marker (MARCM) clones for two known mutant alleles of *fzr*, named *fzr*^A^ and *fzr^B^*, which were isolated by ethyl methanesulfonate (EMS) mutagenesis on X-chromosome (Yamamoto et al., 2014). Both alleles failed to complement *fzr^G0326^*, the hypomorphic allele with a *P{lacZ}* element inserted in the first intron of *fzr*, and were rescued by Dp(1;3)DC120 duplication regarding lethality (this study and Yamamoto et al., 2014). We have tried but failed to characterize the precise lesion of *fzr*^A^ and *fzr*^B^. Whereas all the control type I NSC lineages (*n* = 40) contain a single Dpn^+^ Ase^+^ NSC and 4-5 Dpn^–^ Ase^+^ GMCs that undergo terminal division, *fzr*^A^ and *fzr*^B^ (collectively called *fzr*^–^) mutant clones contain a single NSC but a large number of ectopic GMCs (17.8 ± 6.38 and 19.6 ± 8.20, respectively) (Figures 1A,B). The ectopic GMC phenotype observed in *fzr*^–^ mutants were fully rescued by expressing an UAS- Fzr-HA transgene driven by *tub-Gal4* from MARCM driver as well as by the insertion of Dp(1;3)DC120, a genomic fragment containing the *fzr* locus (Figures 1A,B).

Next, we assessed whether ectopic GMCs were also observed in type II NSC lineages in *fzr*^–^ mutants. There are 8 type II NSC lineages in each larval brain lobe. The defining characteristic of type II NSC clones is that they produce INPs, which can divide 4–6 times to generate GMCs. Within type II lineages, the NSCs can be recognized by their large size (10–14 μm in diameter) and positive for Dpn but negative for Ase. INPs can be identified by their small size (3–4 μm in diameter) and are either Dpn^+^ Ase^+^ (mature INPs) or Dpn^–^Ase^+^ and Dpn^–^ Ase^–^ (immature INPs). We further analyzed the number of immature INPs in *fzr*^–^ type II clones by examining Ets domain-containing transcription factor Pointed P1 (PntP1), which is expressed in both type II NSCs and immature INPs (Zhu et al., 2011). The population of immature INPs are positive for PntP1 and can therefore be distinguished from GMCs. Similar to ectopic GMCs in *fzr*^–^ type I clones, the ectopic Dpn^–^ Ase^+^ cells, which include GMCs and immature INPs, are also observed in *fzr*^–^ mutant type II clones (Supplementary Figures 1A,B). Since we did not detect any change in the number of mature and immature INPs (Supplementary Figures 1C,E–G), ectopic Dpn^–^ Ase^+^ cells observed in type II *fzr*^–^ clones are most likely GMCs. Together, these results suggest that Fzr regulates the homeostasis of GMCs in both type I and type II NSC lineages.

Consistently, the knockdown of *Fzr* in NSCs (and their immediate progenies due to perdurance of Gal4 in progenies) using RNA interference (RNAi) with pan-NSC driver (*wor*-Gal4;UAS-Dcr2) resulted in ectopic GMCs (Figures 1E,F) that are often undergoing cell division (Figures 1E,G). Similarly, the knockdown of *Fzr* specifically in type I or type II NSCs (and immediate progenies of NSCs due to Gal4 protein perdurance) also leads to an increased number of GMCs (Supplementary Figures 1H–K).

Moreover, we also observed ectopic GMCs phenotype in clones from another *fzr*^–^ mutant, *fzr^8F3^* as well as hemizygous mutant *fzr^G0418^*/Y (Figures 1A–D). The EMS *fzr^8F3^* mutant contains a nonsense mutation (Trp214 > Stop) for *fzr* and likely produces short unstable peptides (Silies and Klämbt, 2010). The *fzr^G0418^* has the *P{lacW}* element inserted in the 5′ end of *fzr* gene, and is hypomorphic allele of *fzr* (Jacobs et al., 2002). Besides having ectopic GMCs, a few *fzr*^B^ clones also contained ectopic small Dpn^+^ Ase^+^ NSC-like cells (Figure 1C). Similarly, in *fzr^8F3^* mutant clones, 45.8% (*n* = 24) and 80% (*n* = 20) of mutant type I and type II clones also contained ectopic NSCs (Figures 1A,C and Supplementary Figures 1A,D). While the ectopic GMC phenotype observed in *fzr^8F3^* clone was completely rescued by the insertion of genomic fragment Dp(1;3)DC120, the ectopic NSC phenotype was only partially rescued (Figures 1A,C and Supplementary Figures 1A,D), suggesting an additional background mutation present in the *fzr^8F3^* mutant might partially contribute to the ectopic NSC phenotype. Hereafter we focus on the function of Fzr in regulating GMC population in type I NSC clones.

In conclusion, Fzr plays an important role in NSC lineage progression in developing larval brains and that the Fzr loss results in the formation of ectopic GMCs.

### *Fzr*^–^ Neural Lineages Do Not Display Any Disturbance of NSC Asymmetric Division or Neuronal Dedifferentiation

**FIGURE 2:**
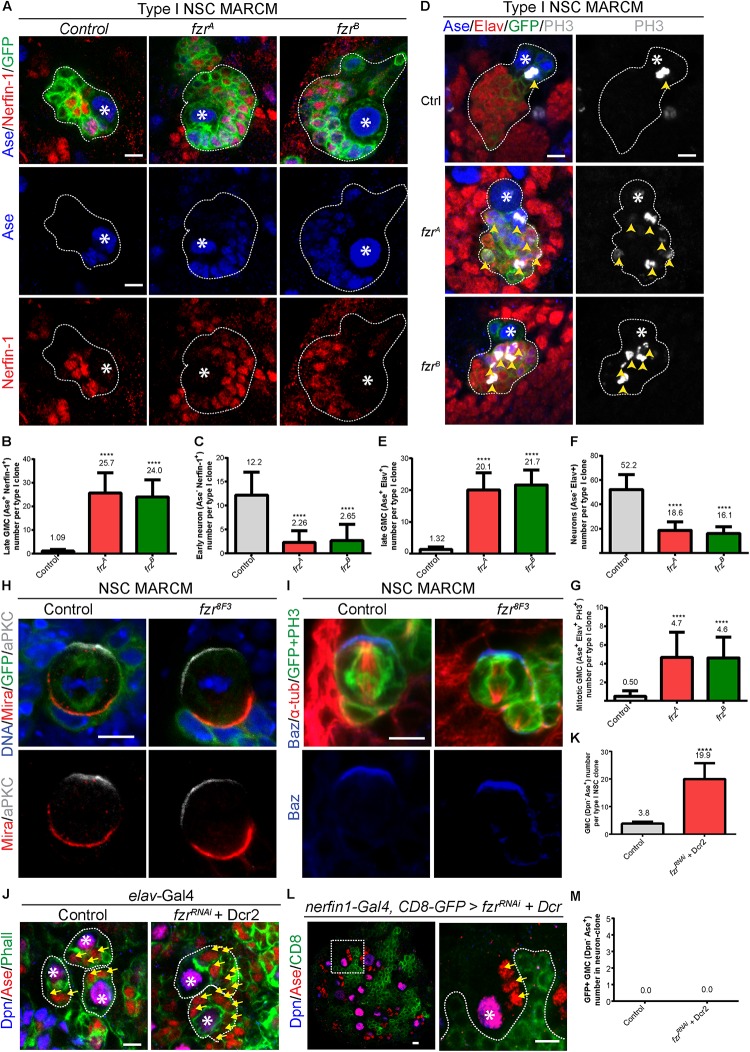
Depletion of Fzr does not impair NSC division nor promote neuronal dedifferentiation. **(A)** Type I MARCM clones of MARCM driver control (FRT19A), *fzr*^A^ and *fzr*^B^ were labeled with Ase, Nerfin-1 and GFP. **(B,C)** Quantifications of the number of Ase^+^ Nerfin-1^+^ cells (**B**, late GMCs) and Ase^–^ Nerfin-1^+^ (**C**, early neurons) for **(A)**. For **(B,C)**, control, *n* = 35; *fzr*^A^, *n* = 23; *fzr*^B^, *n* = 20. **(D)** Type I MARCM clones of MARCM driver control (FRT19A), *fzr*^A^ and *fzr*^B^ were labeled with Ase, Elav, PH3, and GFP. **(E–G)** Quantifications of the number of Ase^+^ Elav^+^ cells **(E)**, Ase*^–^* Elav^+^ (**F**, neurons), and motitic PH3^+^ Ase^+^ Elav^+^ **(G)** for **(D)**. For **(E,G)**, control, *n* = 22; *fzr*^A^, *n* = 21; *fzr*^B^, *n* = 23. For **(F)**, control, *n* = 21; *fzr*^A^, *n* = 20; *fzr*^B^, *n* = 21. **(H)** Mitotic NSCs in control (FRT19A MARCM control) and *fzr^8F3^* MARCM clones (were labeled with DNA, Mira, aPKC, and GFP. Control, *n* = 20; *fzr^8F3^*, *n* = 20. **(I)** Mitotic NSCs in control (FRT19A MARCM control) and *fzr^8F3^* MARCM clones were labeled with Baz, α-tub, PH3, and GFP. Control, *n* = 5; *fzr^8F3^*, *n* = 7. **(J)** Type I NSC lineages of control (β-gal^RNAi^ + Dcr2) or *fzr*^RNAi^ + Dcr2 with pan neuronal driver (*elav*-Gal4) were labeled with Dpn, Ase, and Phall. **(K)** Quantification of the number of Dpn*^–^* Ase^+^ cells (GMCs) for **(J)** control, *n* = 31; *fzr*^RNAi^ + Dcr2, *n* = 15. **(L)** Type I NSC lineages of *fzr*^RNAi^ + UAS-Dcr2 with late-GMC and neuronal driver (*nerfin-1*-Gal4, UAS-CD8-GFP) was labeled with Dpn, Ase and GFP. Enlarged views of the white dotted boxes in the right panel is shown in left panel. **(M)** Quantifications of GMC number found within GFP^+^ neuron-clones for **(L)** control (β-gal^RNAi^ + Dcr2), *n* = 50; *fzr*^RNAi^ + Dcr2, *n* = 50. Data are presented as mean ± SD. ****P ≤ 0.0001. Asterisks, NSCs; yellow arrows, GMCs; yellow arrowheads, mitotic GMCs; white dotted lines, clone outline. Scale bars: 5 μm. n, number of quantified clones. aPKC, atypical protein kinase C; Ase, Asense; α-tub, alpha-tubulin; Baz, Bazooka; Dcr2, Dicer 2; Dpn, Deadpan; Elav, embryonic lethal abnormal visual system; Fzr, Fizzy and cell division cycle 20 related; GMC, Ganglion Mother Cell; MARCM, mosaic analysis with a repressible cell marker; Mira, Miranda; Nerfin-1, Nervous finger 1; NSC, neural stem cell; Phall, Phalloidin; PH3, phospho-Histone H3; UAS, upstream activating sequence.)

To further characterize the cell fate of GMCs accumulated upon the loss of Fzr, we examined the expression of Nerfin-1, which is expressed in late GMCs and newly generated neurons. Whereas each control type I NSC clone contains only on average 1.09 ± 0.702 Ase^+^ Nerfin-1^+^ late GMCs and around 12.2 ± 4.82 Ase^–^ Nerfin-1^+^ early neurons, there were 25.7 ± 8.52 and 24.0 ± 7.31 Ase^+^ Nerfin-1^+^ late GMCs and 2.26 ± 2.47 and 2.65 ± 3.42 Ase^–^ Nerfin-1^+^ early neurons in *fzr*^A^ and *fzr*^B^ mutant clones, respectively (Figures 2A–C). Wild-type neurons express Embryonic lethal abnormal vision (Elav) but not Asense (Figure 2D). Strikingly, we observed a population of Elav^+^ Ase^+^ cells in *fzr*^–^ mutants with a concomitant reduction of Elav^+^ Ase^–^ neurons (Figures 2D–F). Consistently, upon the loss of Fzr in type I MARCM clones, some of the Elav^+^ Ase^+^ cells are undergoing mitosis, as judged by the presence of phosphor-Histone 3 (PH3) (Figures 2D,G). Altogether, these results indicate that loss of Fzr in NSC lineages results in the accumulation of dividing GMCs at the expense of generating neurons undergoing differentiation.

There are three non-mutually exclusive possibilities that could contribute to this interesting phenotype: (1) an ectopic production of GMCs from disrupted asymmetric divisions or extended NSC division; and/or (2) dedifferentiation of neurons to GMCs; and/or (3) failures during GMC differentiation that lead to their ectopic cell divisions.

To address the first possibility that the disturbance in NSC division results in production of excess GMCs, we examined the localization of polarity proteins that are essential for NSC division. We observed that the cellular localization of apically localized aPKC and basally localized Miranda (Mira) in *fzr^8F3^* dividing NSCs are intact and similar to that of the control NSCs (Figure 2H). Similarly, Mira in both control as well as *fzr*^A^ and *fzr*^B^ NSCs are correctly localized (Supplementary Figure 2A). The localization of another apically localized protein Bazooka (Baz, *Drosophila* homolog of mammalian Par3) are also intact in *fzr^8F3^* mutant NSCs (Figure 2I). In addition, the localization of mitotic kinase Polo in dividing NSCs seems unaffected in *fzr*^A^ and *fzr*^B^ NSCs (Supplementary Figure 2B). Besides, the number of progeny cells from a single NSC remains similar upon Fzr loss (Supplementary Figure 1L) and that no elevated cell-death, as judged by the staining of cleaved caspase 3, was observed in *fzr*^A^ clones (Supplementary Figure 2C). Together, these results suggest that NSC division seems normal upon loss of Fzr and that disturbances in NSC division are unlikely the cause of GMC accumulation.

To test whether neuronal dedifferentiation accounts for the ectopic GMCs, we knocked down *Fzr* in different populations of NSCs lineages with *fzr*^RNAi^ with different Gal4 drivers. While the loss of *Fzr* in whole NSC lineage using *elav*-Gal4 or *pros*-Gal4 drivers resulted in ectopic GMCs (Figures 2J,K and Supplementary Figure 2D), the loss of Fzr in late GMCs and early neurons by *nerfin-1*-Gal4 driver did not result in ectopic GMCs within *nerfin-1* expressing clones (labeled by GFP) (Figures 2L,M). Moreover, using an enhancer trap in which beta-galactosidase (β*-Gal*) is inserted at *fzr* locus (*fzr^G0418^*), we did not detect the expression of Fzr in Elav^+^ neurons (Supplementary Figure 2E), suggesting that Fzr does not function in neurons to maintain neuronal differentiation. In summary, our results indicate that neuronal dedifferentiation is unlikely the cause of GMC accumulation.

Interestingly, we observed a high expression of Fzr in glial cells (Supplementary Figure 2F). However, upon the knockdown of Fzr in glial cells by *fzr*^RNAi^ with glial driver (*repo*-Gal4), GMC number remains unchanged (Supplementary Figures 2G–I), indicating that GMC number is not regulated by Fzr in glial cells.

In summary, our results suggest that ectopic GMC accumulation upon the loss of Fzr is likely caused by the failed differentiation of GMCs that leads to ectopic GMC divisions.

### Fzr^–^ Neural Lineages Display Defects in GMC-to-Neuron Transition

**FIGURE 3:**
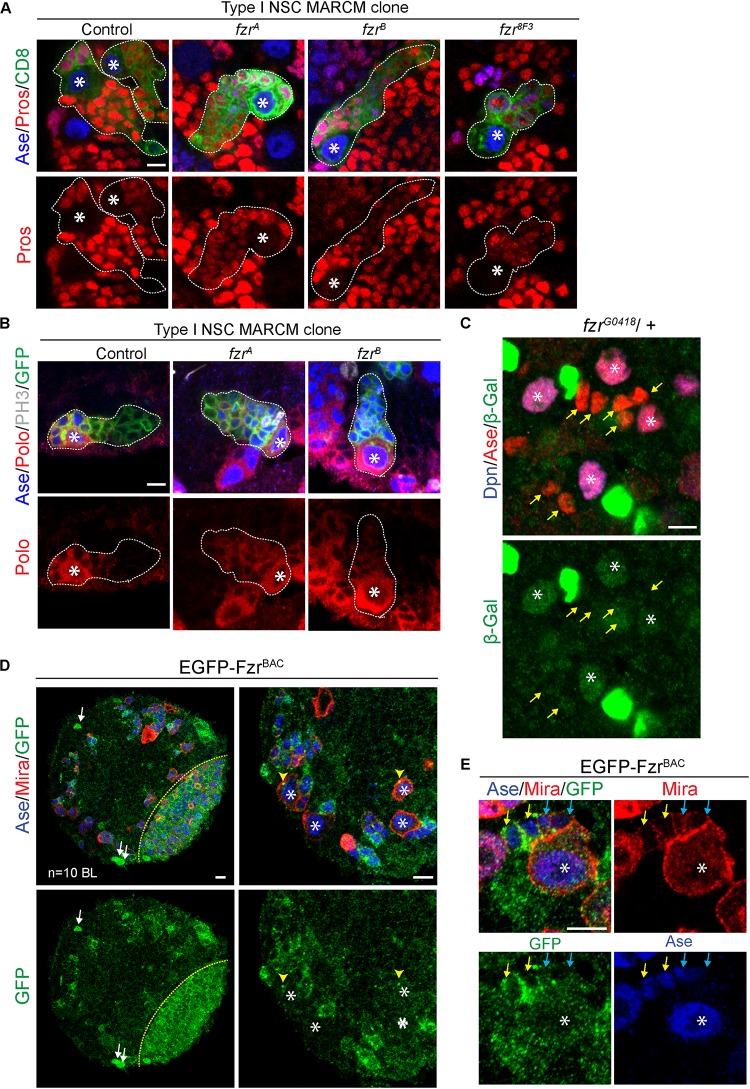
Fzr promotes the transition from GMCs to neurons. **(A)** Type I MARCM clones of MARCM driver control (FRT19A), *fzr*^A^, *fzr^B^*, and *fzr^8F3^* were labeled with Ase, Pros, and CD8. Control, *n* = 22; *fzr*^A^, *n* = 32; *fzr*^B^, *n* = 27; *fzr^8F3^*, *n* = 20. **(B)** Type I MARCM clones of MARCM driver control (FRT19A), *fzr*^A^ and *fzr*^B^ were labeled with Ase, Polo, PH3, and GFP. Control, *n* = 30; *fzr*^A^, *n* = 50; *fzr*^B^, *n* = 50. **(C)** NSC lineages in third instar larval brain of heterozygous *fzr^G0418^* (*fzr*-*lacZ*)/ + were labeled with Dpn, Ase, and β-Gal, product of *lacZ* gene that is inserted in *fzr* locus. *n* = 10 brain lobes. Yellow arrows, GMCs. **(D,E)** Larval brains expressing genomic EGFP-Fzr^BAC^ were co-stained with Ase and Mira. *n* = 10 brain lobes. Yellow arrows, late GMCs; blue arrows, early GMCs; white arrows, glial like cells; yellow arrowhead, centrosome-like punctate; yellow dotted lines indicate boundary between the optic lobe and the central brain. Asterisks, NSCs; white dotted lines, clone outline. Scale bars: 5 μm. Ase, Asense; β-gal, β-galactosidase; Dcr2, Dicer 2; Dpn, Deadpan; EGFP, enhanced green fluorescent protein; Fzr, Fizzy and cell division cycle 20 related; GFP, green fluorescent protein; GMC, Ganglion Mother Cell; MARCM, mosaic analysis with a repressible cell marker; Mira, Miranda; NSC, neural stem cell; PH3, phospho-Histone H3; Polo, Polo kinase; Pros, Prospero.

To test if there are any defects during the transition of GMCs to neurons, we assessed the expression of differentiation factor Prospero (Pros) (Chu-Lagraff et al., 1991; Choksi et al., 2006) in *fzr*^–^ NSC clones. In most *fzr*^–^ clones, the expression of Pros is substantially reduced in comparison to those of control clones (Figure 3A). Moreover, the expression of Polo kinase, which promotes cell division and stemness (Sunkel and Glover, 1988; Glover, 2005), remained high in ectopic GMCs of *fzr*^–^ clones (Figure 3B). All these results suggest that the ectopic mutant GMCs maintain their undifferentiated states and are unable to differentiate into neurons.

In addition, Fzr displayed moderate staining in wild-type NSCs and weak staining in GMCs (Figure 3C, asterisks and yellow arrows, respectively), consistent with our model that Fzr functions within GMCs to promote the transition of GMCs into neurons. To further characterize the expression pattern of endogenous Fzr in the nervous system, we attempted to generate anti-Fzr antibodies for immunostaining without success (data not shown). Next, we turned to labeling Fzr protein with enhanced GFP (EGFP) within its endogenous locus, hereafter called EGFP-Fzr^BAC^. Consistent with the localization of Fzr shown by the enhancer trap, EGFP-Fzr^BAC^ displayed strong staining in glial cells, as judged by their glial morphology and cortical location (Figure 3D, white arrows) as well as being positive for the glial marker Repo (Supplementary Figure 3A). Besides, EGFP-Fzr^BAC^ also displayed strong localization in late  GMCs (Figure 3E, yellow arrows), minimal level in early GMCs (Figure 3E, blue arrows, as judged by their small size, being Ase^+^ Mira^weak^ cells, and their immediate proximity to the big Ase^+^ Mira^+^ type I NSCs) and weak level in NSCs (Figure 3E, white asterisks).

Moreover, Fzr is known for its function as an activator of the ubiquitin ligase complex APC/C to promote the ubiquitination and degradation of various substrates during G1 (Sigrist and Lehner, 1997; Jacobs et al., 2002; Schaeffer et al., 2004). We wondered whether Fzr functions in NSC lineages might be mediated by APC/C^Fzr^ complex. To test this, we knocked down *ida*, a subunit of APC/C, in type II NSC driver. However, upon *ida* knockdown, no ectopic GMCs was observed, although other defects such as loss of NSCs, loss of NSC identity and mitotic arrest were observed (Supplementary Figure 3B). These phenotypes were also observed upon knockdown of *cdc20*, the co-activator of APC/C during mitosis (Supplementary Figure 3B). These phenotypes were consistent with known functions of APC/C in dividing cells and in agreement with the mitotic defects reported for *ida* and *cdc20* mutants (Dawson et al., 1993; Bentley et al., 2002), indicating the RNAi-mediated knockdown were efficient. Furthermore, upon the overexpression of HA tagged Rca1, the negative regulator of Fzr-dependence APC/C activity (Grosskortenhaus and Sprenger, 2002), no ectopic GMCs were observed in both type I and type II lineages (Supplementary Figures 3D–H), although HA-Rca1 protein was successfully over-expressed in type II NSCs and their immediate progenies (Supplementary Figure 3C). These results highlight a possibility that Fzr might function independently of APC/C in regulating GMC-to-neuron transition. Because the loss of function of APC/C blocks the mitotic progression of NSCs to generate GMCs, we are unable to completely rule out the potential involvement of APC/C in GMC-to-neuron differentiation.

### *Fzr* Loss Enhances Ectopic Type II NSCs Resulted From Downregulation of Differentiation Factors *Erm* or *Brm*

**FIGURE 4:**
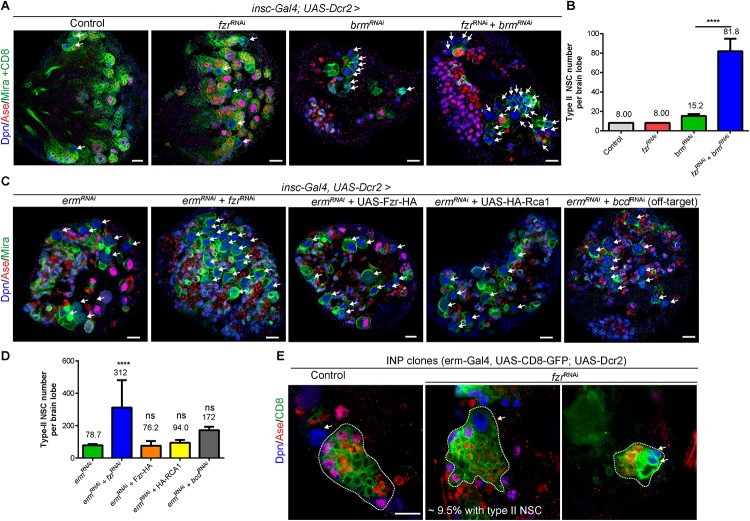
Fzr genetically interacts with Brm and Erm to regulate NSC-INP transition. **(A)** Larval brains of control (β-gal^RNAi^ + UAS-CD8-GFP), *fzr*^RNAi^ (+ UAS-CD8-GFP), *brm*^RNAi^ (+ β-gal^RNAi^) or *fzr*^RNAi^ + *brm*^RNAi^ with insc-Gal4; UAS-Dcr2 were labeled with Dpn, Ase, Mira, and CD8. Note that to balance the number of UAS elements across different genotypes, additional control UAS line, β*-gal*^RNAi^ or UAS-CD8-GFP, were added to various RNAi lines, resulting in weaker phenotype in RNAi lines compared with those without additional UAS control shown earlier in this study. **(B)** Quantifications of type II NSC number per brain lobes for **(A)**. Control, *n* = 10; *fzr*^RNAi^, *n* = 10; *brm*^RNAi^, *n* = 5; and *fzr*^RNAi^ + *brm*^RNAi^, *n* = 6. **(C)** Larval brains of *erm*^RNAi^ (+ β-gal^RNAi^), *fzr*^RNAi^ + *erm*^RNAi^, *erm*^RNAi^ + Fzr-HA, *erm*^RNAi^ + HA-RCA1 or *bcd*^RNAi^ + *erm*^RNAi^ with *insc*-Gal4, UAS-Dcr2 were labeled with Dpn, Ase, and Mira. **(D)** Quantification of type II NSC number per brain lobes for **(C)**. *erm*^RNAi^, n = 11; *fzr*^RNAi^ + *erm*^RNAi^, *n* = 20, *erm*^RNAi^ + Fzr-HA, *n* = 21; *erm*^RNAi^ + HA-RCA1, *n* = 10 and *bcd*^RNAi^ + *erm*^RNAi^, *n* = 10. **(E)** INP clones of driver control (β-gal^RNAi^) or *fzr*^RNAi^ under control of INP driver (*erm*-Gal4, UAS-CD8-GFP; UAS-Dcr2) were labeled with Dpn, Ase, and CD8. Control, *n* = 90; *fzr*^RNAi^, *n* = 105. White dotted lines, INP clone outline. The middle and right panels displayed two different clones of *fzr*^RNAi^ under the control of INP driver. Respectively, one large and several small type-II NSCs were found within INP clones. Data are presented as mean ± SD. ****P ≤ 0.0001; ns for *P* > 0.05. White arrows, type II NSCs (∼10–12 μm in diameter and Dpn^+^ Ase*^–^*). Scale bars: 10 μm. Ase, Asense; β-gal, β-galactosidase; Bcd, bicoid; Brm, Brahma; Dcr2, Dicer 2; Dpn, Deadpan; Erm, Earmuff; Fzr, Fizzy and cell division cycle 20 related; GFP, green fluorescent protein; GMC, Ganglion Mother Cell; INP, intermediate neural progenitor; Insc, Inscuteable; Mira, Miranda; ns, statistically non-significant; NSC, neural stem cell; UAS, upstream activating sequence.

Our data shows that the loss of Fzr also results in the formation of ectopic NSCs in addition to excess GMCs (Figures 1A–C and Supplementary Figures 1A–D). Brahma (Brm) and Earmuff (Erm) are important to suppress dedifferentiation of INPs, and the loss of either *Brm* or *Erm* results in the reversion of INP to type II NSCs. Next we wondered if *Fzr* genetically interacted with *Brm* or *Erm* to suppress INP dedifferentiation. While *Fzr* knockdown alone using NSC-specific driver did not cause ectopic type II NSCs (8 type II NSCs per brain lobe similar to a control brain lobe, Figures 4A,B), the loss of *Brm* resulted in ectopic type II NSCs (15.2 ± 1.92 NSC, *n* = 5). However, knockdown of *Fzr* dramatically enhanced dedifferentiation defects associated with *Brm* loss to 81.8 ± 12.8 NSCs per brain lobe (Figures 4A,B). Similarly, the knockdown of *Fzr* using NSC-specific driver (*insc-Gal4*) substantially enhanced the ectopic type II NSCs of *Erm* knockdown (Figures 4C,D, 78.7 ± 7.30 NSCs, *n* = 11 BLs in *erm*^RNAi^ + control^ RNAi^ vs. 311.9 ± 169.6 NSCs, *n* = 20 BLs in *erm*^RNAi^ + *fzr*^RNAi^). The overexpression of *Rca1*, negative regulator of Fzr-dependent functions of APC/C complex, as well as *Fzr* overexpression did not affect the severity of Erm loss, suggesting Fzr might function independent of APC/C complex in the regulation of INP-NSC homeostasis (Figures 4C,D). Interestingly, the knockdown of *Fzr* using INP driver *(erm-Gal4, UAS-CD8-GFP)* resulted in ectopic type II NSCs from 9.5% of INP clones (*n* = 105), while none of the control INP clones contained any type II NSCs (Figure 4E). Due to the unavailability of independent RNAi lines targeting different *fzr* sequences and the lethality of homozygous *fzr* mutants in the early embryonic or larval stages, we were unable to confirm genetic relationship of *Fzr* and *Brm* or *Erm* in NSC-INP transition with additional RNAi lines or mutants. Moreover, knockdown of the potential off-target of *fzr^RNAi^ (GD#25553)*, *bicoid* (*bcd*), by RNAi under the control of the same NSC- specific driver *(insc-Gal4)* did not lead to as strong enhancement of INP dedifferentiation associated with *Erm* loss, in comparison to the effect of *fzr*^RNAi^ (Figures 4C,D), suggesting that loss of *fzr* partially contributed to the genetic enhancement observed in the double knockdown *fzr*^RNAi^ and *erm*^RNAi^.

Altogether, these results suggest that beside its roles in regulating GMC-to-neuron transition, *Fzr* genetically interacts with *Brm* and *Erm* to promote NSC-INP transition.

## Discussion

**FIGURE 5:**
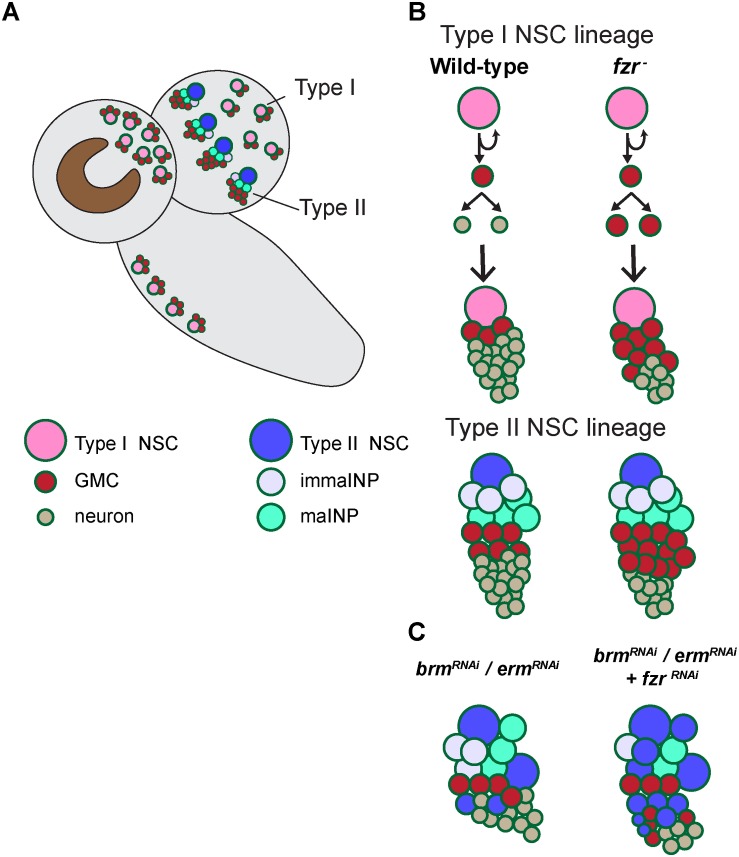
A working model illustrating the mechanism by which Fzr promotes GMC-neuron differentiation. **(A)** A presentation of larval central nervous system with type-I and type-II NSC lineages. **(B)** In *wild-type Drosophila* larval brains, the neural progenitor cells called GMCs are generated from the division of NSCs or intermediate neural progenitor cells INPs in type I and type II lineages, respectively. GMCs then divide once to generate differentiated neurons or glial cells. Loss of *fzr* in NSC lineages impairs GMC-to neuron differentiation and thus the accumulation of GMCs at the expense of neurons. **(C)** Knockdown of the INP maturation factors *Brm* or *Erm* results in ectopic NSCs originating from the dedifferentiated INPs in type-II NSC lineages. *Fzr* loss enhances the ectopic type II NSCs induced by *Brm* or *Erm* downregulation.

Here, we show that Fzr/Cdh1, an established co-activator of APC/C ubiquitin ligase known for its roles in regulating cell cycle or post-mitotic functions in terminally differentiated neurons or glial cells, promotes the commitment of neural progenitor GMCs to the production of terminally differentiated neurons (Figures 5A,B). In this study, we present multiple lines of evidence to support the function of Fzr in regulating GMC-to-neuron differentiation: (1) the presence of Fzr in late GMCs; (2) the accumulation of GMCs at the expense of neurons in *fzr*^–^ mutant NSC clones; and (3) the loss of differentiation factor Pros and ectopic accumulation of mitotic Polo kinase in NSC progenies upon loss of *fzr*. Beside functioning in GMCs, Fzr is also present in low level in NSCs to regulate NSC-to-INP transition of type II NSC lineages (Figures 5A,C).

In this study, we observed a strong expression of EGFP-Fzr in the optic lobe (Figure 3D). However, the function of Fzr in optic lobes is currently unclear. Recently, the APC/C^Fzr^ complex has been reported to promote retinal differentiation in *Drosophila* eye imaginal discs and thus the formation of adult eyes (Martins et al., 2017). In eye discs, Fzr modulates Wingless Signaling via Nek2 degradation, which is essential for progenitor cells to differentiate. As such, it would be interesting to examine if Fzr might regulate GMC differentiation in NSC lineages of the central brain through a similar mechanism.

In this study, we provide putative evidence that *Fzr* interacts with *Erm* and *Brm* to regulate NSC-INP balance. Interestingly, Ets2, the mammalian homolog of the master regulator of type II lineages PntP1, is stabilized upon Cdh1 deficiency and is proposed to be substrate of APC/C^Cdh1^ complex (Li et al., 2008). However, it remains to be determined how *Fzr, Erm*, or *Brm* cooperate to regulate NSC-INP transition in type II NSC lineages and if the underlining mechanisms for Fzr’s function in GMC-neuron and NSC-INP transitions are shared.

In mammals, Cdh1/Fzr and several core components of APC/C complex are highly expressed in post-mitotic neurons (Gieffers et al., 1999) and Cdh1/Fzr participates in the regulation of neuronal axonal and dendritic growth, synapses, metabolism and survival of neurons (Eguren et al., 2011). Besides, Fzr/Cdh1 functions to prevent replicative stress and p53-dependent cell death in neural progenitors (Eguren et al., 2013). In this study, we uncover a new role of Fzr in *Drosophila* nervous system: ensuring the commitment of neural progenitors to differentiation cascade. Large scale human cancer tissue arrays and prognostic analyses have indicated that loss of APC^Cdh1^ function correlates with various human carcinogenesis, including brain tumors (reviewed in Qiao et al., 2010). Besides, inactivation of Cdh1 has been implicated in excitation-mediated neuronal cell death in neurological disorders such as Alzheimer disease (Maestre et al., 2008). Our study on the role of Fzr in turning off proliferation in neuronal cells may provide insight into how Fzr deficit may contribute to human neurodegenerative diseases and tumors.

## Data Availability Statement

All datasets generated for this study are included in the article/Supplementary Material (see Supplementary Data Sheet S2 for the numerical data used in this study).

## Author Contributions

PL: conceptualization, data curation, formal analysis, investigation, methodology, writing – the original draft, and writing – review and editing. HW: conceptualization, funding acquisition, project administration, supervision, writing – review and editing the original draft, and writing – review and editing.

## Conflict of Interest

The authors declare that the research was conducted in the absence of any commercial or financial relationships that could be construed as a potential conflict of interest.

## Abbreviations

APC/C: anaphase promoting complex/cyclosome
aPKC: atypical protein kinase C
Ase: asense
α-tub: alpha-tubulin
BAC: bacterial artificial chromosome
Baz: bazooka
BDSC: Bloomington Drosophila Stock Center
Bcd: bicoid
β-gal: β-galactosidase
Brm: Brahma
Cdc20: cell division cycle 20
Dcr2: Dicer 2
Dpn: deadpan
Elav: embryonic lethal abnormal visual system
EGFP: enhanced green fluorescent protein
Erm: Earmuff
Fzr: fizzy and cell division cycle 20 related
GFP: green fluorescent protein
GMC: Ganglion Mother Cell
Ida: imaginal discs arrested
INP: intermediate neural progenitor
Insc: inscuteable
Lola: longitudinals lacking
MARCM: mosaic analysis with a repressible cell marker
Mira: Miranda
Nerfin-1: nervous fingers 1
NSC: neural stem cell
ns: statistically non-significant
Phall: phalloidin
PH3: phospho-Histone H3
Polo: polo kinase
Pros: prospero
Repo: reversed polarity
Rca1: regulator of cyclin A1
RNAi: RNA interference
UAS: upstream activating sequence
VDRC: Vienna Drosophila Resource Center
Wor: Worniu.
