# Supplementary material for: Fzr/Cdh1 Promotes the Differentiation of Neural Stem Cell Lineages in Drosophila
Source: Front Cell Dev Biol. 2020 Feb 11;8:60. doi: 10.3389/fcell.2020.00060 (PMC7026481; doi:10.3389/fcell.2020.00060)
Supplement: Supplementary file 1 [file Data_Sheet_1.PDF]

Supplementary Material

1 Supplementary Figures and Tables

1.1 Supplementary Figures

Supplementary Figure 1. Related to Figure 1. Ectopic GMCs are generated upon Fzr loss in type I and type II NSC lineages.

(A) Type II MARCM clones of MARCM driver control (FRT19A), *fzr*^{A}, *fzr*^{B}, *fzr*^{8F3}, *fzr*^{A} + *tub>Fzr-HA*, *fzr*^{B} + *tub>Fzr-HA* and *fzr*^{8F3} + *tub>Fzr-HA* were labeled with Dpn, Ase and CD8. *tub>Fzr-HA* refers to UAS-Fzr-HA driven by *tub-Gal4* from MARCM driver, so Fzr-HA is expressed only in MARCM clones that lose *tub-Gal80* upon mitotic recombination.

(B, C and D) Quantifications of the number of Dpn^{-} Ase^{+} cells (B, immature INPs and GMCs), Dpn^{+} Ase^{+} (C, mature INPs) per type II MARCM clones and the percentage of clones with two or more Dpn^{+} Ase^{-} NSCs (D) for (A). Control, n = 11; *fzr*^{A}, n = 24; *fzr*^{B}, n = 22; *fzr*^{8F3}, n = 20; *fzr*^{A} + *tub>Fzr-HA*, n = 21; *fzr*^{B} + *tub>Fzr-HA* , n = 17; *fzr*^{8F3} + *tub>Fzr-HA* , n = 19.

(E) Type II MARCM clones of MARCM driver control (FRT19A), *fzr*^{A} and *fzr*^{B} were labeled with Ase, PntP1 and CD8.

(F and G) Quantifications of the number of PntP^{+} Ase^{-} (F) and PntP^{+} Ase^{+} immature INPs (G) for (E). For (E and F), control, n = 22; *fzr*^{A}, n = 25; *fzr*^{B}, n = 20.

(H) Type I NSC lineages of control (*β-gal*^{RNAi}) or *fzr*^{RNAi} with Type I NSC driver (*ase-Gal4; UAS-mCD8-GFP*) were labeled with Dpn, Ase and CD8.

(I) Type II NSC lineages of control (*β-gal*^{RNAi}) or *fzr*^{RNAi} with Type II NSC driver (*wor-Gal4, ase-Gal80/CyO; UAS-mCD8-GFP*) were labeled with Dpn, Ase and CD8.

(J and K) Quantifications of the number of Dpn^{-} Ase^{+} cells (J, immature INPs and GMCs) and Dpn^{+} Ase^{+} (K, mature INPs) for (I). For both (J) and (K), control, n = 5; *fzr*^{RNAi}, n = 10.

(L) Cell number per type I or type II MARCM clones in MARCM driver control (FRT19A), *fzr*^{A} and *fzr*^{B}. For type I clones: control, n = 22; *fzr*^{A}, n = 28; *fzr*^{B}, n = 20. For type II clones: control, n = 20; *fzr*^{A}, n = 24; *fzr*^{B}, n = 21.

Data are presented as mean ± SD. ** for $0.001<P \leq 0.01$; **** for $P \leq 0.0001$. Asterisks, NSCs; white arrowheads, GMCs (and immature Ase^{+} Dpn^{-} INPs for Fig S1A and S1I); white arrows, Ase^{-} PntP1^{+} immature INPs; blue arrows, Ase^{+} PntP1^{+} immature INPs; white dotted lines, clone outline. Scale bars, 5µm. n, number of quantified clones.

Ase, Asense; Dcr2, Dicer 2; Dpn, Deadpan; Fzr, Fizzy and cell division cycle 20 related; GMC, Ganglion Mother Cell; INP, intermediate neural progenitor; MARCM, mosaic analysis with a repressible cell marker; Mira, Miranda; ns, statistically nonsignificant; NSC, neural stem cell; PntP1, Pointed isoform P1; UAS, upstream activating sequence; Wor; worniu.

Supplementary Figure 2. Related to Figure 2. Fzr promotes NSC lineage differentiation independent on NSC asymmetric division or its functions in glial cells.

(A) Metaphase NSCs in control (FRT19A), *fzr*^{A} and *fzr*^{B} MARCM clones were labeled with DNA, Mira, PH3 and GFP. n = 10 for each genotype.

(B) Metaphase NSCs in control (FRT19A), *fzr*^{A} and *fzr*^{B} MARCM clones were labeled with Ase, Polo, PH3 and GFP. control, n = 10; *fzr*^{A}, n = 11; *fzr*^{B}, n = 10. White arrowheads, kinetochore localization of Polo; yellow arrowheads, mitotic spindle pole localization of Polo.

(C) Type I MARCM clones of MARCM driver control (FRT19A) and *fzr*^{A} were labeled with Mira, cleaved caspase-3 and GFP. n = 20 for each genotype. Asterisks, NSCs; white dotted lines, clone outline.

(D) Type I NSC lineages on the ventral side of third instar larval brain lobes of control (*β-gal*^{RNAi} + *UAS-Dcr2)* or *fzr*^{RNAi} + *UAS-Dcr2* with pan-neural driver (*pros*-Gal4) were labeled with Dpn, Ase and Phall. n = 20 brain lobes for each genotype.

(E) NSC lineages in third instar larval brain of heterozygous *fzr*^{G0418} (*fzr-lacZ*) /+ were labeled with DNA, Elav, and β-Gal (the product of *lacZ* gene that is inserted in *fzr* locus). n = 10 brain lobes. Asterisks, NSCs.

(F) The larval brain lobe of heterozygous *fzr*^{G0418}/+ were labeled with DNA, Repo, and β-Gal. White arrowheads, Repo^{+} β-Gal^{+} glial cells. n = 10 brain lobes.

(G) Type I NSC lineages on the ventral side of third instar larval brain lobes of control (*β-gal*^{RNAi} + *UAS-Dcr2*) or *fzr*^{RNAi} + *UAS-Dcr2* with glial driver (*repo-Gal4*).

(H and I) Quantification of the number of Dpn^{-} Ase^{+} cells (GMCs, H) and Dpn^{+} Ase^{+} (NSCs, I) for type I NSC lineages in (G). For (H), control, n = 20 clones; *fzr*^{RNAi} + *UAS-Dcr2*, n = 27 clones. For (I), control, n = 5 brain lobes; *fzr*^{RNAi} + *UAS-Dcr2*, n = 5 brain lobes.

Data are presented as mean ± SD. * for P ≤ 0.05 and ns for P > 0.5. Scale bars, 20 μm (D, F and G) and 5 μm (the rest).

Ase, Asense; β-gal, β-galactosidase; Dcr2, Dicer 2; Dpn, Deadpan; Elav, embryonic lethal abnormal visual system; Fzr, Fizzy and cell division cycle 20 related; GMC, Ganglion Mother Cell; MARCM, mosaic analysis with a repressible cell marker; Mira, Miranda; NSC, neural stem cell; Phall, Phalloidin; PH3, phospho-Histone H3; Polo, Polo kinase; Pros: Prospero; Repo: reversed polarity; UAS, upstream activating sequence.

Supplementary Figure 3. Related to Figure 3. Fzr localization in larval brain.

(A) Larval brains expressing genomic EGFP-Fzr^{BAC} were co-stained with Dpn and Repo. n = 10 brain lobes. White arrows, Repo^{+} Fzr^{+} glial cells.

(B) Type II NSC lineages of control (β-gal^{RNAi}), *ida*^{RNAi}, *cdc20*^{RNAi_1} and *cdc20*$^{RNAi\_2}$ with Type II NSC driver (*wor-Gal4, ase-Gal80; UAS-CD8-GFP*) were labeled with Dpn, Ase and CD8.

(C) Larval brain lobes of *UAS-HA-RCA1* with Type II NSC driver (*wor-Gal4, ase-Gal80; UAS-CD8-GFP*) were labeled with HA and CD8. The right panel is the zoom-in image of the area outlined by the yellow dotted box in the left panel.

(D) Larval brain lobes of control (*β-gal*^{RNAi}) or *UAS-HA-RCA1* with NSC driver (*wor*-Gal4) were labeled with Dpn, Ase and Phall. Yellow dotted lines indicate boundary between optic lobes and central brains.

(E) Quantifications of the number of Dpn^- Ase^+ cells (GMCs) per type I clones for (D). control, n = 30; *UAS-HA-RCA1*, n = 51.

(F) Type II NSC lineages of control (*β-gal*^{RNAi}) or *UAS-HA-Rca1* with Type II NSC driver (*wor-Gal4, ase-Gal80; UAS-mCD8-GFP)* were labeled with Dpn, Ase and CD8.

(G and H) Quantifications of the number of Dpn^- Ase^+ cells (G, immature INPs and GMCs) and Dpn^+ Ase^+ (H, mature INPs) for (F). Control, n = 4; UAS-HA-Rca1, n = 18.

Data are presented as mean ± SD. ns with P > 0.05. Asterisks, NSCs; white dotted lines, clone outline. Scale bars, 10 µm (A and D), 5 µm (the rest).

Ase, Asense; β-gal, β-galactosidase; Cdc20, cell division cycle 20; Dcr2, Dicer 2; Dpn, Deadpan; EGFP, enhanced green fluorescent protein; Fzr, Fizzy and cell division cycle 20 related; GFP, green fluorescent protein; GMC, Ganglion Mother Cell; Ida, Imaginal discs arrested; INP, Intermediate neural progenitor; ns, statistically nonsignificant; NSC, neural stem cell; Rca1, Regulator of cyclin A1; Repo: reversed polarity; UAS, upstream activating sequence; Wor; Worniu.

1.2 Supplementary Table 1

Table 1. The primers used for generation of EGFP-Fzr^{BAC}

Name	Sequence	Note
Fzr-N-tag-EGFP-F	GCA AGT TTT GTT TGG TTA CAT TTG AGT TTG TGT TGA GTT TTT GCC AGC CAA AGG CGC TTA AGA TGA TGG TGA GCA AGG GCG AGG AG	generation of recombineering EGFP-Fzr^{BAC}
Fzr-N-tag-EGFP-PL452R	GAT TCC GTG CCA CAG GAC TGT AGT GCT TCA GGA TGC GCT TCT CGT ACT CGG GAC TAA AAC TAG TGG ATC CCC TCG AGG GAC	
Fzr-N-reco-F	AGTCCGTCGAAAAACAGCAC	To verify EGFP-Fzr^{BAC}
Fzr-N-reco-R	ATAACGGCTCGTGCAGAGTT	
Fzr-N-reco2-F	GTC GCT GTA GTC CGT CGA AAA ACA GCA C	
Fzr-N-reco2-R	ATA ACG GCT CGT GCA GAG TTC AAT GC	
