# Supplementary figures and images for: Fzr/Cdh1 Promotes the Differentiation of Neural Stem Cell Lineages in Drosophila
Source: Front Cell Dev Biol. 2020 Feb 11;8:60. doi: 10.3389/fcell.2020.00060 (PMC7026481; doi:10.3389/fcell.2020.00060)

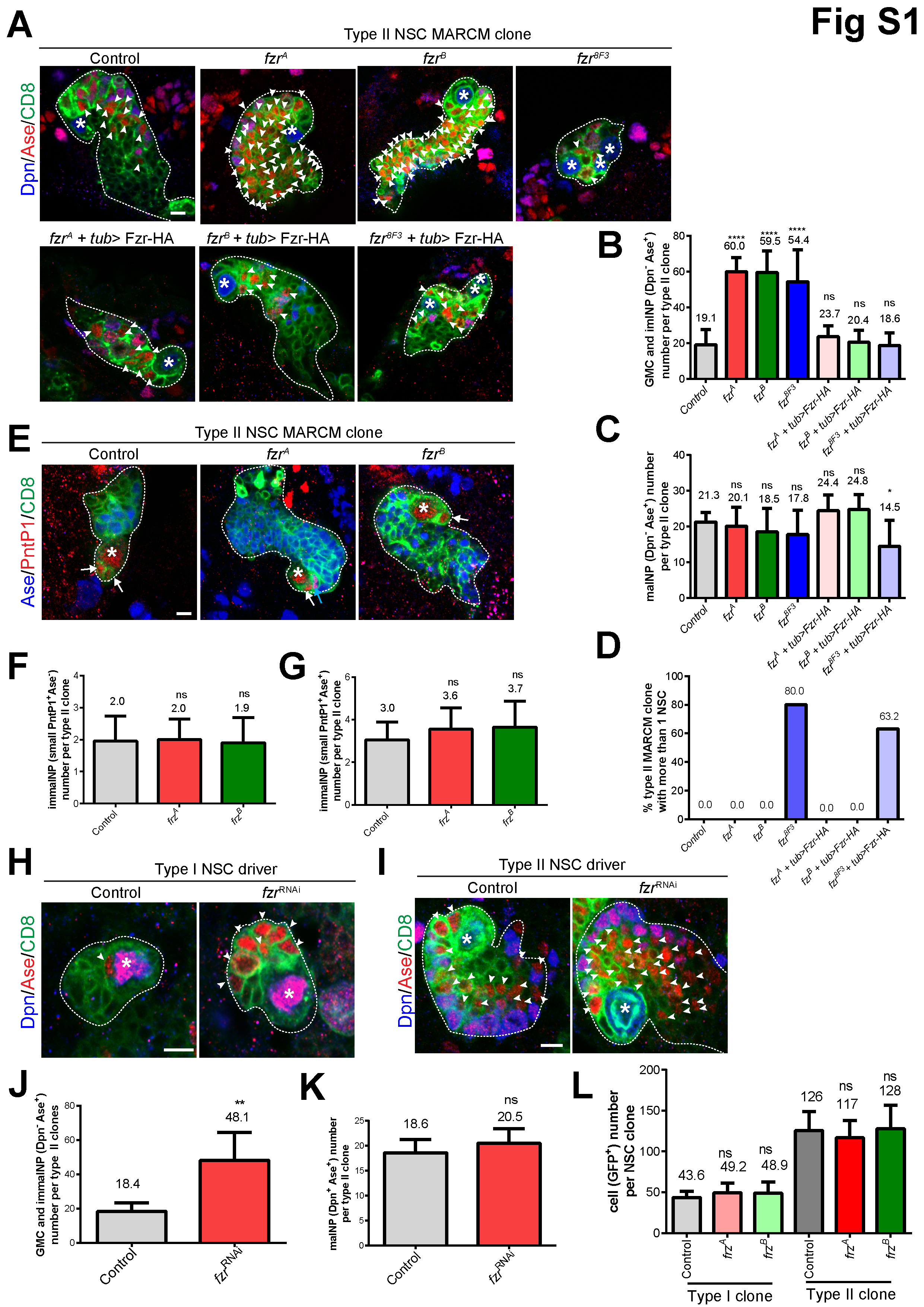

Supplement: Supplementary file 3 [file Image_1.jpg]

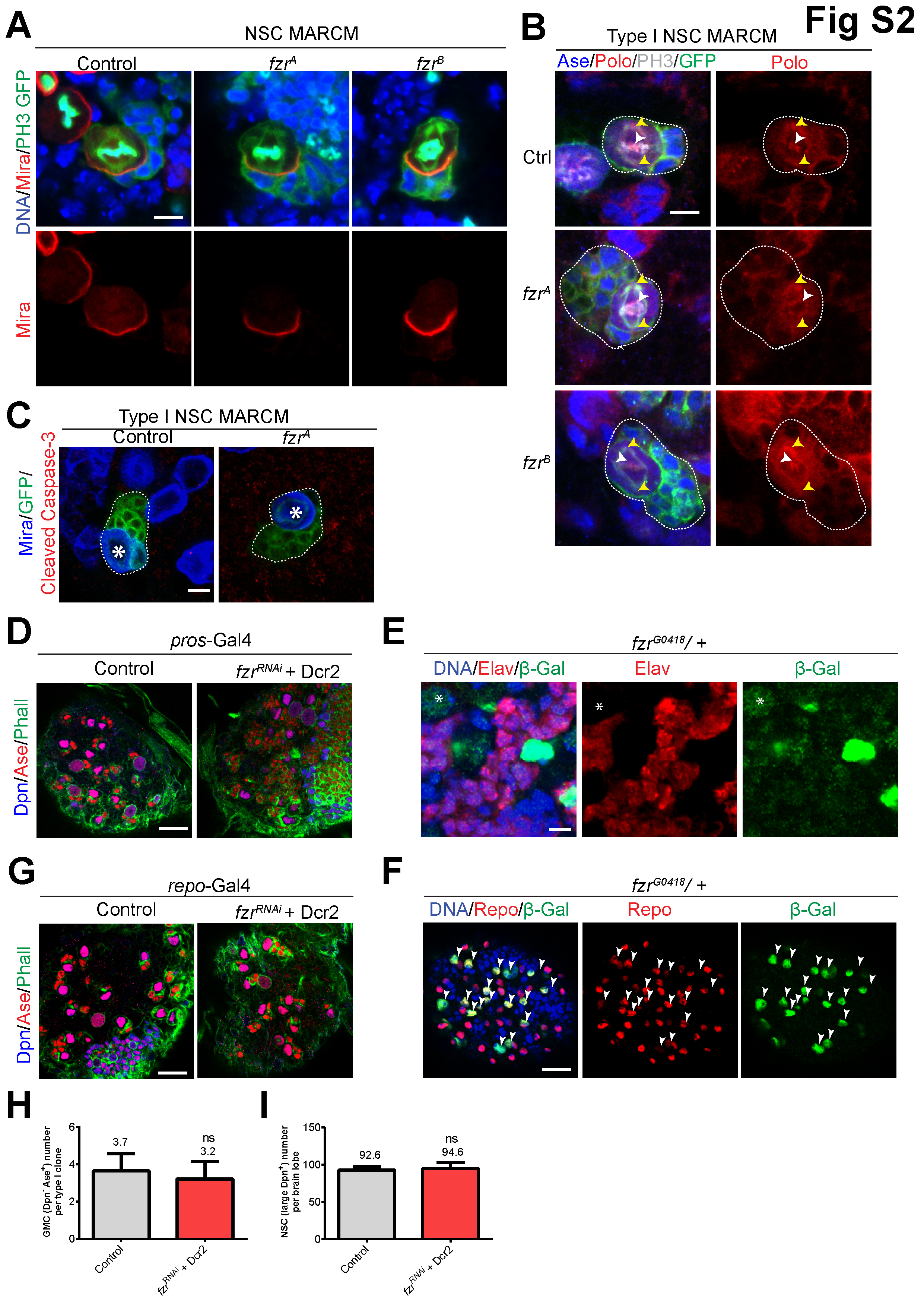

Supplement: Supplementary file 4 [file Image_2.jpg]

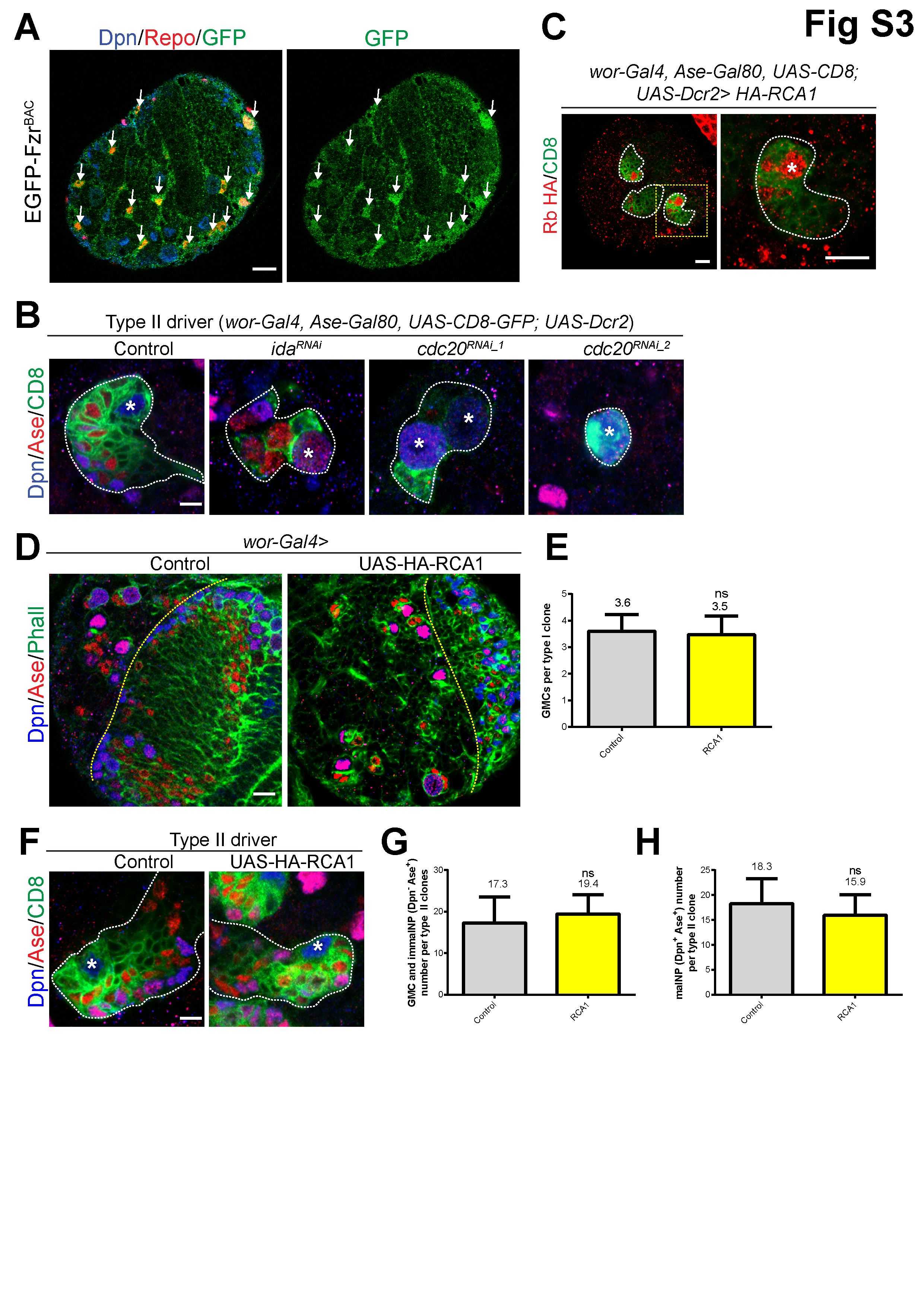

Supplement: Supplementary file 5 [file Image_3.jpg]
